# Emergency laparoscopy – current best practice

**DOI:** 10.1186/1749-7922-1-24

**Published:** 2006-08-31

**Authors:** Oliver Warren, James Kinross, Paraskevas Paraskeva, Ara Darzi

**Affiliations:** 1 Department of BioSurgery and Surgical Technology, Imperial College of Science, Technology and Medicine, St. Mary's Hospital, London, UK

## Abstract

Emergency laparoscopic surgery allows both the evaluation of acute abdominal pain and the treatment of many common acute abdominal disorders. This review critically evaluates the current evidence base for the use of laparoscopy, both diagnostic and interventional, in the emergency abdomen, and provides guidance for surgeons as to current best practise. Laparoscopic surgery is firmly established as the best intervention in acute appendicitis, acute cholecystitis and most gynaecological emergencies but requires further randomised controlled trials to definitively establish its role in other conditions.

## Background

The emergence of laparoscopy in the late 1980's as a credible therapeutic intervention heralded a new surgical age. Demonstrable reduction of wound complications, post-operative pain, hospital stay and costs in treating gallbladder disease [1] and gynaecological conditions such as laparoscopic sterilisation [2] and hysterectomy [3] led to the expansion of its use in other abdominal organ pathology, such as the colon [4], stomach [5] and oesophagus [6]. Initially laparoscopy was limited to elective surgery but as technology and surgical experience expanded so did the application of laparoscopy into the emergency setting [7]. Laparoscopic surgery has now been described in many abdominal emergencies, such as acute appendicitis [8], blunt and penetrating trauma [9], perforated peptic ulcer disease [10] and acute pancreatitis [11], and this variety of conditions seems set to expand further.

When considering the role of emergency laparoscopy there are two distinct clinical scenarios that need to be considered. The first is that a specific pathology is assumed following diagnostic workup and thus a specific procedure is planned, the second is that abdominal pathology of uncertain causation or severity is present, and thus the primary aim of laparoscopy will be diagnostic. Over the last twenty years or more, a number of large cohort studies have reported high definitive diagnosis rates of between 86–100% in unselected patients [12-14], and as surgical experience and technology have improved so have the number of patients who are subsequently managed exclusively with laparoscopic surgery [15,16]. Emergency diagnostic laparoscopy is not without distracting arguments; missed diagnoses, procedure related complications and delay to definitive open surgical procedure are all potential negatives.

This review aims to critically evaluate and summarise the current evidence base for the use of laparoscopy, both diagnostic and interventional, in the emergency abdomen, and to guide surgeons as to current best practise. We wish to emphasise that any endorsement for a laparoscopic approach is only valid for surgical units with experience and sufficient expertise in minimal access surgery.

## Trauma

Prior to modern day ultrasonography (US) and computed tomography (CT) scanning, laparotomy for abdominal trauma was negative and non-therapeutic in approximately one-third of cases [17], leading to increased morbidity and cost [18]. However the use of abdominal helical and/or triple-contrast CT to evaluate abdominal injuries have substantially reduced this figure to around 6% [19]. Despite being first reported in the mid-1970's [20,21] laparoscopy for the diagnosis and treatment of traumatic abdominal injuries remains a relatively ill-defined concept. Only two randomized studies have reported on laparoscopy in trauma [22,23] but despite this paucity of data some recommendations can be made. It would appear that laparoscopy in trauma has a role in well-selected patients, who, primarily, must be haemodynamically stable, because in unstable patients emergency surgical exploration of the abdomen may be life saving.

### Diagnostic laparoscopy

A significant number of patients who sustain penetrating trauma to the anterior abdominal wall do not suffer a peritoneal breach [24]. Proving that penetration has not occurred negates the need for laparotomy, but current diagnostic modalities, including US and CT scanning are unable to do this due to high false – negative rates. Laparoscopy has been shown to be highly effective at determining peritoneal penetration [25,26], resulting in decreased laparotomy rates [23], length of stay [27] and cost [28].

Laparoscopy is an excellent modality in the evaluation of the diaphragm in penetrating thoracoabdominal injuries. Current imaging modalities are limited because of low sensitivity [29], as is DPL. Laparoscopy provides direct visualisation of the left diaphragm and more limited visualization of the right diaphragm, and if found to be intact, laparotomy may be avoided [9]. Both of the randomized control trials assessing laparoscopy in trauma patients focused on its diagnostic properties.

Whilst stable patients with blunt abdominal trauma may undergo diagnostic laparoscopy to exclude relevant injury, it's utility in this sub-group of patients is still relatively unproven [30].

### Therapeutic laparoscopy

The use of therapeutic laparoscopy remains controversial, with the majority of the literature compromising case reports or series. Laparoscopic repair of perforating injuries to the diaphragm represents the most frequently described therapeutic application [31-33] but there are increasing reports of laparoscopic haemostasis of minor injuries to the liver or spleen [34,35] and therapeutic use of laparoscopy to repair limited gastrointestinal injuries [36]. Some surgeons advocate interval washout of intra-peritoneal blood [37] or bile [38] following visceral injury to decrease ileus and peritoneal symptoms, and in two isolated reports to cell-salvage blood for autologous transfusion [37,39]. Despite promising results in these reports, the paucity of clear trial-based data prohibits specific recommendations regarding the therapeutic use of laparoscopy in trauma victims.

## Perforated peptic ulcer disease

Peptic ulcer perforation is the second most frequent abdominal perforation requiring surgery [40] and accounts for 5% of abdominal emergencies [41]. Laparoscopic repair of a perforated peptic ulcer was first reported in 1990 [42] but the technique has yet to be universally accepted. Two large high quality randomized studies have been performed comparing laparoscopic to open surgical repair [43,44], involving in total 214 patients (111 in the laparoscopy group and 103 in the open group). The first study found no benefits in the laparoscopic group in terms of total hospital stay, time to resume normal diet, morbidity, reoperation or mortality rates [43]. The second reported patients in the laparoscopic group to suffer significantly less postoperative pain and have a shorter operating time [44]. A recent meta-analysis of these two studies concluded that there is also a trend to a decrease in septic abdominal complications with laparoscopic surgery [40]. Further comparative studies have described a reduction in postoperative complication rates after laparoscopic surgery, but may be biased by patient selection [45-49]. Laparoscopic patients did however experience less post operative pain in the medium to long term, which may account for the shorter hospital stay, and earlier return to normal activities. Mortality may also be marginally lower in those treated laparoscopically [50]. Laparoscopic repair has a conversion-to-open rate of 10–20% and furthermore, revision surgery is more frequently required after laparoscopic surgery than in open cases [51,52]. There is currently no comparative evidence from a systematic review to suggest whether a primary, patch repair or fibrin sealing is the most effective method of repair when administered laparoscopically. Although the recent European Association of Endoscopic Surgeons' Consensus Statement states that laparoscopy is 'clearly superior' for patients with perforated peptic ulcer disease [30], we believe that more randomised control trials are required before this statement can be fully supported.

## Acute cholecystitis

There is little role for laparoscopy in the diagnosis of acute cholecystitis. Acute cholecystitis can be diagnosed with near 100 % specificity from a combination of clinical features, ultrasound findings, and a white cell count >10 × 10/L or a CRP >100 mg/dL [53]. Over 48,000 cholecystectomies were performed in the UK in 2004–05 [54] and a laparoscopic approach is now generally considered to be the gold standard for this procedure. Several published studies have compared open cholecystectomy to laparoscopic cholecystectomy for acute cholecystitis [55-59]. However only two of these were randomised [55,56]. All demonstrated a faster recovery, and a shorter hospital stay in the laparoscopic treated group.

The key question in current practice regarding laparoscopic cholecystectomy is not 'if' but 'when'? Initially a delayed approach was favoured, due to fear of complications in the immediate setting, a longer operation time and a higher conversion rate. However with greater experience and an improvement in surgical skills [60-62], recent randomised control trials have suggested this is not necessarily the case [63,64]. A recent meta-analysis of RCT data by Lau reported reduced conversion-to-open rates (16% vs. 23%), blood loss, cost and length of hospital stay in the early group [65]. However operation time and complication rates were comparable for the two groups and there was significantly less bile leakage after delayed laparoscopic surgery. Furthermore we feel the conversion-to-open rates to be unacceptably high in both groups. One convincing argument for immediate intervention appears to be the failure of initially conservative management, where up to one fifth of patients fail to improve and ultimately require acute surgery [66,67]. Of the remaining 80%, 29% were subsequently readmitted with recurrent episodes before their planned surgery, adding to cost and morbidity.

In conclusion, we believe all patients with acute cholecystitis should be offered a laparoscopic cholecystectomy within 72 hours of the initial diagnosis, if economic and local workforce restrictions allow.

## Appendicectomy

Appendicitis is a common diagnosis, with approximately 8% of the US population undergoing appendicectomy during their lifetime [68]. Due to the non-specific nature of its presentation negative appendicectomies are still a common occurrence. This suggests a potential role for laparoscopy, as both a diagnostic tool that allows good visualisation of the right iliac fossa, and a route for therapeutic intervention.

The laparoscopic appendicectomy (LA) versus open appendicectomy (OA) question is one that has been extensively investigated. Over fifty randomised studies exist in the literature, and numerous systematic reviews have been undertaken [69-72]. The most recent systematic review examined 54 randomised studies with a total population of 5000 patients. Whilst heterogeneity was high between some studies, it seems wound infection rates are about half as likely in LA but a post-operative intra-abdominal collection is nearly three times more likely [70]. This study reported a reduction of 1.1 days in hospital stay which is similar to a database review of over 40,000 cases that demonstrated a reduction of 0.8 days [73].

Among the many randomised studies comparing LA and OA, only a few studies have explicitly used the findings of a diagnostic laparoscopy to guide the subsequent surgery. Most are in female patients of fertile age, and document significant reductions in the numbers of negative appendicectomies, and rate of unestablished diagnoses [74,75]. The diagnostic advantages in men and children are less clear due to the relative ease of diagnosis in these sub-groups.

In cases where a separate pathology is found, there is good evidence to suggest a normal appendix should be left in-situ [76]. What remains unclear is whether to remove a normal appendix in patients with an otherwise unremarkable laparoscopy. Contributory to this is the reliability of the macroscopic diagnosis of appendicitis [77,78] and the potential morbidity associated with three port sites is greater. There is not enough evidence currently to rule for or against removing the normal appendix in this scenario.

When compared to the traditional OA, laparoscopy is more expensive and it requires specific expertise [70]. However, the EAES advise that patients with symptoms and signs of acute appendicitis should undergo a diagnostic laparoscopy and appendicectomy [30] and we feel that there is enough evidence to support this statement in the setting of appropriate surgical expertise.

Finally the usage of emergency laparoscopy in the paediatric arena has been limited both by local experience and modification and availability of paediatric equipment. The area where there is evidence is the emergency management of appendicitis in children. A recent meta-analysis by Aziz et al compared laparoscopic with open appendicectomy, looking at endpoints such as post-operative ileus, wound infection, post operative pyrexia, and intra-abdominal abscess formation [79]. In addition, parameters such as operative time and length of stay were examined. The study suggested that the laparoscopic approach to appendicectomy was associated with reduced complications however higher quality randomised trials would be required to confirm this. Also this approach in children is not as widely accepted as it has been in adults.

## Gynaecological disorders

Many acute gynaecological disorders can be diagnosed and treated via laparoscopy [79]. In gynaecological emergencies CT scanning is rarely helpful, and usually a combination of pregnancy testing, clinical acumen and trans-vaginal (TV) and trans-abdominal (TA) US scanning are utilised to formulate a differential diagnosis. Following these conventional investigations, diagnostic laparoscopy is highly effective [80] and recommended [81].

There is a significant amount of high quality evidence regarding the role of laparoscopic surgery in ectopic pregnancy (EP). In confirmed EP, laparoscopy should be performed unless haemodynamic instability is present. It is fast, cheaper [82], and fertility outcome is comparable to laparotomy [83]. Furthermore, hospitalization and sick leave times are shorter, and adhesion development reduced when compared to laparotomy [84]. If tubal rupture has occurred, a laparoscopic salpingectomy should be performed. However, in cases of unruptured tubal pregnancy, a tube preserving operation should be considered [85].

Ovarian cyst torsion is an organ threatening condition that causes patients to present with acute lower abdominal pain. Initially, pregnancy must be excluded, and a TV US scan performed to exclude ovarian cyst formation. If pain fails to settle, a laparoscopy must be performed to exclude adnexal torsion [30]. Any ovarian cysts found during laparoscopy can be treated laparoscopically [86]. Laparoscopic surgery to repair ovarian torsion is superior to open [87] and is suitable even in pregnancy.

Salpingo-oophoritis commonly causes acute pelvic and lower abdominal pain, and can mimic other surgical diagnoses. Diagnostic laparoscopy can be useful to exclude other common pathologies. If the diagnosis is correct, microbiological samples can be taken to target anti-microbial therapy, and in pyosalpinx, pus can be drained laparoscopically [88].

In conclusion, if gynaecological disorders are the suspected cause of pain, diagnostic laparoscopy should be performed, as frequently simultaneous therapy will be possible.

## Mesenteric ischemia

Acute mesenteric ischemia is due to arterial occlusion (approximately 50% of cases), venous occlusion (15%) and non-occlusive mesenteric ischemia (35%). Clinical diagnosis is usually confirmed by the use of selective mesenteric angiography or CT scanning [89,90]. One of the most important factors determining the prognosis of these patients is early and prompt diagnosis [91]. Depending on the duration and extent of ischemia treatment consists of embolectomy, or laparotomy with resection of infarcted bowel segments in cases where the patient develops signs of peritonitis. The potential role of emergency laparoscopy therefore in this condition relates to it diagnostic rather than its therapeutic opportunities.

Certain benefits of diagnostic laparoscopy are suggested. These patients are frequently severely dehydrated and acidotic, with significant co-morbidity, and as such are at significant risk from contrast-dependent angiography. Conversely diagnostic laparoscopy is relatively quick and well tolerated and if necessary can be performed at the bedside in the Intensive Care Unit or Emergency room [92,93]. At laparoscopy the small and large bowel can be visualised and other conditions causing an acute abdomen may be diagnosed enabling correct management. However, the rate of mesenteric ischemia among patients with an acute abdomen is only 1% [94], and laparoscopy does not guarantee correct recognition of mesenteric ischemia particularly in early cases. Nor does it allow palpation of the small bowel mesentery to detect arterial pulsation. Despite therefore a few published case reports advocating its use [95,96], we suggest that in cases of suspected mesenteric ischemia, clinical assessment combined with conventional imaging remains the best way to assess the need for intervention.

## Acute pancreatitis

There are many causes of acute pancreatitis but gallstones and excess alcohol consumption are by far the commonest [97]. Similarly, there is a large spectrum of clinical presentation and thus assessment of severity is key to successful management. However, laparoscopy for diagnostic or prognostic reasons is unnecessary, as this is obtainable through clinical presentation, appropriate imaging [98,99], and severity scores such as the Imrie score, the APACHE II score [100] and Ranson's Criteria [101].

The surgical management of acute pancreatitis is heavily dependent upon the aetiology and severity of the episode. Unless there is an urgent indication such as haemorrhage or abdominal compartment syndrome, surgery should be delayed until the patient is adequately resuscitated and there is sufficient demarcation of any necrosis that may develop [102,103]. If acute surgical exploration is unavoidable, laparoscopic surgery has been advocated for exploration, irrigation, drainage and necrosectomy [104-106] but in the absence of any high quality evidence, the open approach remains the gold standard [107]. If necrosis has organised, dependent upon it's type and location, three laparoscopic operative approaches have been reported: infracolic debridement [108], retroperitoneal debridement [109] and transgastric pancreatic necrosectomy [110]. Whilst no randomized studies performed, infracolic debridement has been most favourably reported, with patient survival of 85% [111]. Acute pancreatitis, or an acute exacerbation of chronic pancreatitis can lead to pseudocyst formation. Internal drainage is indicated 6 weeks after the first documentation of a pseudocyst and this can be performed laparoscopically. Laparoscopic pseudocyst gastrostomy, cyst jejunostomy, or cyst duodenostomy may all be indicated, depending on the size and location of the lesion [11].

In gallstone pancreatitis, bile duct clearance and cholecystectomy are essential to prevent disease recurrence. Thus all patients with biliary pancreatitis should undergo definitive management of their gallstones during the same hospital admission or at the very least, within two weeks [30,112]. In mild cases, the best approach to this is laparoscopic cholecystectomy with intraoperative cholangiography, as opposed to postoperative Endoscopic Retrograde CholangioPancreatography (ERCP) [113]. However, intraoperative laparoscopic bile duct exploration requires a significant amount of surgical expertise, and if this is not available pre-operative bile duct clearance must be ensured, either by ERCP or Magnetic Resonance CholangioPancreatography (MRCP). MRCP allows detection of choledocholithiasis with sensitivity and specificity bother over 90% [114], and thus in most patients a clear preoperative MRCP is enough to avert intra-operative bile duct exploration.

Finally, in patients with predicted or actual severe gallstone pancreatitis, or when there is cholangitis, jaundice, or a dilated common bile duct, surgery is contraindicated. In this situation an urgent ERCP with endoscopic sphincterotomy should be performed, followed by interval laparoscopic cholecystectomy when the patient is fitter [115-117].

## Acute diverticulitis

Acute diverticulitis is easily diagnosed with a combination of clinical evidence, blood count, inflammatory markers and CT scanning. CT scanning is an excellent modality for the assessment of severity and perforation [118], and as such there is no role for diagnostic laparoscopy in this condition.

Laparoscopic resection of the diseased portion of colon should be avoided in the emergency setting, since the rate of conversion to open and rate of primary re-anastamosis depend on the presence and severity of acute inflammation. The value of elective laparoscopic surgery for diverticular disease is promising, but requires further randomized control trials to fully evaluate its potential [119]. Laparoscopic surgery has been utilised in the setting of diverticular perforation with associated peritonitis (Hinchey Classification III and IV). In patients who are high risk, a laparoscopic approach may be used for exploration and peritoneal lavage [120], or the placement of an omental patch [121]. Associated abscesses can be drained laparoscopically [122]. However, expert centres have reported these cases, and generally it is too early to recommend laparoscopic emergency surgery in diverticular disease.

## Incarcerated hernia

The evidence for the use of laparoscopic surgery in herniorrhaphy (inguinal, incisional and others) is excellent, but the majority of studies exclude emergency cases. It is not safe practise to simply transfer the impressive results from the elective setting and presume they support the use of laparoscopic surgery in the management of incarcerated herniae. To our knowledge there are no randomised control trials comparing open versus laparoscopic surgery for emergency herniorrhaphy. The largest case series reported by Leibl et al showed similar results to elective groin hernia repairs, but the authors were all highly experienced laparoscopic surgeons [123]. In the absence of any comparative studies investigating open versus laparoscopic repair of incarcerated herniae, the open approach must remain the standard treatment.

## Small bowel obstruction

Emergency surgery is a necessity when small bowel obstruction fails to resolve after a period of conservative management, or where urgent decompression of the bowel is required. Laparoscopic treatment of acute bowel obstruction was first reported in 1991 [124], but except for one retrospective matched-pair analysis [125] there are no comparative studies to assess the potential benefits of laparoscopic surgery. Furthermore, this study found a significantly higher rate of iatrogenic bowel perforation in the study group compared to conventional open surgery. Purported benefits of the laparoscopic approach include faster recovery of bowel motility and shorter hospital stay.

Many series have reported that complete laparoscopic treatment appears possible only in around 50% of cases [126-128], patients frequently being converted to open so as to deal with malignancy, bowel perforation and other problems. This has led some groups to attempt to define predictive factors for conversion; a history of two or more surgical abdominal operations, late operation (> 24 hours post-onset), and a bowel diameter exceeding 4 cm have all been reported [129,130].

In conclusion, whilst initial diagnosis and cautious adhesiolysis can be performed at laparoscopy, this must be performed using an open access technique [30], and in most patients, and for most surgeons, open surgery remains the most appropriate intervention.

## Non-specific abdominal pain

Whilst most patients presenting with abdominal pain will be diagnosed within a short period of assessment, there remains a cohort in whom the clinical picture remains equivocal. In these patients, depending on the severity of their symptoms, laparoscopy plays a role. If patient's symptoms and laboratory findings are relatively less concerning, then a period of observation may allow clarification of a diagnosis or simply cessation of the pain. However in those where observation is not safe, due to the severity of the clinical findings, we advocate diagnostic laparoscopy. The reasons for this are two fold. Firstly, converted-to-open cases have a similar outcome to primary laparotomy, thus minimising potential negative effects of laparoscopy [131]. Secondly, as discussed above, many common pathologies, which may be the underlying cause of the non-specific abdominal pain, are now best managed laparoscopically.

## Discussion

As we have outlined above, the available evidence demonstrates a clear advantage to diagnostic and therapeutic laparoscopy in certain common conditions. However, laparoscopy cannot currently be justified in other scenarios until further research (in the form of large randomised trials if possible) is carried out. Our current recommendations are summarised in Table 1.

**Table 1:** Summary of our recommendations regarding the emergency use of laparoscopy.

***CONDITION***	***DIAGNOSTIC ROLE?***	***THERAPEUTIC ROLE?***	***SPECIFICS***
**Trauma**	Penetrating Trauma to Abdominal Wall	Diaphragmatic injury repair	Cautious recommendations
	Evaluation of potential diaphragmatic injuries	Haemostasis of minor visceral injury	
**Perforated PUD**	No	Probably	More research required
**Acute Cholecystitis**	No	Yes	Within 72 hours of presentation
**Appendicitis**	Yes (females) Unclear (Males and Children)	Yes	To be left in-situ if other pathology found
**Gynaecological Emergencies**	Yes	Yes	Ectopic Pregnancy
			Ovarian Cyst Torsion
			Salpingo-Oophritis
**Pancreatitis**	No	No	Immediate Surgery
		Yes	Necrosectomy and pseudocyst drainage
		Immediate Lap. Cholecystectomy.	Mild Gallstone Pancreatitis
		Delayed Lap. Chole after urgent ERCP	Severe Gallstone Pancreatitis
**Mesenteric Ischaemia**	No	No	-
**Acute Diverticulitis**	No	No	Perhaps, *in extremis *where patient is too ill for laparotomy
**Incarcerated Hernia**	No	No	-
**Small Bowel Obstruction**	No	No	-
**Non-Specific Abdominal Pain**	Yes	Yes	-

One limitation of our review is that we have not discussed the use of emergency laparoscopy in the paediatric population. This is for two reasons. Firstly the pathological spectrum in the young is significantly different to the adult population and we feel should be addressed in a separate article. Secondly, we are not a paediatric unit, and have significantly less experience in this patient demographic. We are aware however that emergency laparoscopy can be a useful tool in older children and adolescents [69,132].

Future research must concentrate on those pathologies in which only limited evidence currently exists, and must be multi-centred, not just based in highly specialised units. This will become easier as laparoscopic expertise becomes more mainstream.

Laparoscopic surgery has improved our management of surgical emergencies and in certain conditions is now an essential part of our armamentarium. What is clear is that as surgical expertise and technology both continue to improve, so the remit for laparoscopic surgery will expand, to the benefit of our patients.

## Authors' contributions

OW and JK performed the literature search and extracted the data. OW, JK and PP all wrote different subsections of the review. AD conceived of the review, and edited the manuscript. All authors read and approved the final manuscript.
